# Structural and Functional Characterization of Recombinant Interleukin-10 from Indian Major Carp *Labeo rohita*

**DOI:** 10.1155/2016/3962596

**Published:** 2016-09-01

**Authors:** Sweta Karan, Pujarini Dash, Himani Kaushik, Pramoda K. Sahoo, Lalit C. Garg, Aparna Dixit

**Affiliations:** ^1^Gene Regulation Laboratory, National Institute of Immunology, Aruna Asaf Ali Marg, New Delhi 110067, India; ^2^School of Biotechnology, Jawaharlal Nehru University, New Delhi 110067, India; ^3^Fish Health Management Division, Central Institute of Freshwater Aquaculture, Kausalyaganga, Bhubaneswar 751 002, India

## Abstract

Interleukin-10, an important regulator of both the innate and adaptive immune systems, is a multifunctional major cytokine. Though it is one of the major cytokines, IL-10 from the Indian major carp,* Labeo rohita,* has not yet been characterized. In the present study, we report large scale production and purification of biologically active recombinant IL-10 of* L. rohita* (r*Lr*IL-10) using a heterologous expression system and its biophysical and functional characterization. High yield (~70 mg/L) of soluble r*Lr*IL-10 was obtained at shake flask level. The r*Lr*IL-10 was found to exist as a dimer. Far-UV CD spectroscopy showed presence of predominantly alpha helices. The tertiary structure of the purified r*Lr*IL-10 was verified by fluorescence spectroscopy. Two-dimensional gel analysis revealed the presence of six isoforms of the r*Lr*IL-10. The r*Lr*IL-10 was biologically active and its administration significantly reduced serum proinflammatory cytokines, namely, interleukin 1*β*, TNF*α*, and IL-8, and augmented the NKEF transcript levels in spleen of* L. rohita*. Anti-inflammatory role of the r*Lr*IL-10 was further established by inhibition of phagocytosis using NBT reduction assay* in vitro. *The data indicate that the dimeric alpha helical structure and function of IL-10 of* L. rohita *as a key regulator of anti-inflammatory response have remained conserved during evolution.

## 1. Introduction

Innate and adaptive immune systems have not been extensively explored in fish immunology. The tight link between the innate and adaptive immune systems is governed by signaling molecules such as cytokines, transcription factors, and several innate receptors. Cytokines significantly modulate the inflammatory process, which plays an important protective role in innate immunity. Interleukin-10 (IL-10), initially known as cytokine synthesis inhibitory factor, is a pleiotropic cytokine and demonstrates dominant immunosuppressive function [1]. IL-10 is produced from variety of cells, including T cells, B cells, neutrophils, eosinophils, epithelial cells, keratinocytes, mesangial cells, monocytes/macrophages, NK cells, and tumor cells [2]. IL-10 gene has been cloned and characterized from several vertebrates such as human, mouse, chicken, bottlenose dolphin, pekin duck, turkey, seabass, grass carp, zebrafish, common carp, goldfish, and rainbow trout [3–14].

The IL-10 has been reported to downregulate IL-1, TNF*α*, and IL-6 expression* in vivo,* whereas it stimulates NK cells resulting in antibody-dependent cell mediated cytotoxicity [15]. Oxidative stress has also been reported to be modulated by IL-10 as it suppresses reactive oxygen intermediate species generation [16, 17]. IL-10 suppresses inflammatory responses elicited by activated macrophages, inhibits nitric oxide production, downregulates major histocompatibility complex (MHC) class II expression, inhibits synthesis of a number of macrophage-derived proinflammatory factors such as IL-1*β*, tumor necrosis factor alpha (TNF*α*), IL-12, and cyclooxygenase-2, and thereby downregulates the host immune response to invading pathogens [18–20]. In mammals, different activities of IL-10 appear to be mediated through formation of oligomeric complexes of IL-10 with its receptors expressed on the cell surface [21]. Homodimeric structure of IL-10 has been demonstrated in other species such as gold fish and seabass [9, 13]. Differential expression of IL-10 in different tissues of a number of fish species upon infection with microorganisms or stimulation with LPS indicates its role in immunomodulation in fishes as well [11, 12, 14, 22–25]. Immunoregulatory function of IL-10 has been demonstrated in several fish species as well. Recombinant IL-10 from gold fish significantly reduced the expression of proinflammatory cytokines in monocytes activated with heat-killed* Aeromonas salmonicida*, whereas it stimulated the expression of suppressor of cytokine signaling-3 (SOCS-3) in these cells [13]. Similarly, carp IL-10 downregulated oxygen radical and nitrogen radical production by both macrophages and neutrophils and upregulated SOCS-3 expression [26]. These investigators demonstrated that carp IL-10 stimulated a subset of CD8^+^ memory T cells and downregulated CD4^+^ memory Th1 and Th2 responses. In grass carp also, the IL-10 upregulated the cellular activity of peripheral blood lymphocytes and played an essential role in TGF-*β*1 mediated immune regulation [10]. Thus, IL-10 is one of the major cytokines which may affect the disease status of vertebrates including fishes.

Indian major carp* Labeo rohita* belongs to the carp family Cyprinidae and is commercially one of the most important and highly favored fish in the Indian subcontinent. The fishery products are valuable source of animal protein and essential micronutrients. Fishery sector is vulnerable to adverse impacts of diseases and environmental conditions. A number of gram negative bacteria have been associated with fish pathogenic conditions [27–29]. Changes in the levels of cytokines during infectious stage play a vital role in immune suppression and disease progression. It is important to understand the mechanisms involved in the protection against many of these pathogens. Since fish suffer from many bacterial and viral infections, which result in major economic losses, it is imperative to understand their immune system to control infection. Cytokines are widely used as adjuvants in mammals, whereas in fish their possible use as vaccine adjuvants is minimally explored. Understanding of biochemical and functional characteristics of various cytokines of fish may also pave the way for their use to develop strategies for controlling infections.

In the present study, we report high level expression and characterization of IL-10 of* L. rohita* and its effect on expression profile of immune related genes in* L. rohita*.

## 2. Materials and Methods

### 2.1. Construction of Histidine-Tagged r*Lr*IL-10 cDNA Clones

For recombinant expression in* Escherichia coli*, synthetic cDNA corresponding to* Lr*IL-10 gene was designed according to codon preference in* E. coli. Nde*I and* Xho*I restriction sites were included in the synthetic cDNA at the 5′ and 3′ ends, respectively, for convenient cloning in the expression vector, pET22b(+) (Novagen, USA). Recombinant pUC57-*Lr*IL-10 harboring the synthetic codon biased* Lr*IL-10 gene fragment of 483 nucleotides (including the restriction sites, encoding the mature* L. rohita* IL-10 of 157 amino acid residues) was obtained from GenScript, USA. The gene fragment encoding the mature r*Lr*IL-10 was excised out from pUC57-*Lr*IL-10 plasmid using* Nde*I and* Xho*I (NEB, USA) and was further cloned into pET22b(+) (Novagen, USA) digested with same enzymes. Putative recombinants designated as pET.*Lr*IL-10 were analyzed by restriction enzyme digestion and confirmed by automated DNA sequencing (DNA sequencing facility, Department of Biochemistry, University of Delhi South Campus, New Delhi). The expressed product from this construct would be of 163 amino acid residues including a 6x histidine tag at the C-terminus of the protein.

### 2.2. Expression of 6x Histidine-Tagged r*Lr*IL-10 in* E. coli*

For expression and localization analysis of r*Lr*IL-10 protein, plasmid pET.*Lr*IL-10 was transformed into* E. coli *BL21 (*λ*DE3) cells. Induction of expression from the recombinants was achieved essentially as described earlier [30]. Briefly, the transformed cells were grown in Luria-Bertani broth (Difco) containing 100 *μ*g/mL ampicillin under continuous shaking (220 rpm) at 37°C until optical density reached 0.6. Recombinant protein expression was induced by addition of 1 mM isopropyl 1-thio-*β*-D-galactopyranoside (IPTG) and the cultures were then allowed to grow further for 5 h. Cells were harvested by centrifugation at 3000 ×g for 5 min at 4°C and resuspended in 10 mM Tris-HCl, pH 7.0, in the absence or presence of 0.5 M arginine and lysed by sonication (5 pulses of 1 s for 40 cycles). The cell lysates were centrifuged at 12,000 ×g for 20 min at 4°C. The soluble and insoluble fractions separated by centrifugation were analyzed in SDS-PAGE (12%) by coomassie brilliant blue (CBB-R250) staining.

### 2.3. Purification of Soluble r*Lr*IL-10

The soluble r*Lr*IL-10 protein was purified using Hispur Cobalt resin (Thermo Scientific, USA) as per the manufacturer's instructions. Briefly, the solubilized r*Lr*IL-10 was mixed with the resin and left for overnight binding with end-to-end shaking at 4°C. The slurry was centrifuged at 1200 ×g in a swinging-bucket rotor for 2 min. The flow through was discarded and nonspecifically bound proteins were removed by washing the resin twice in wash buffer (10 mM Tris-HCl, pH 8.0, and 10 mM imidazole). The r*Lr*IL-10 protein was eluted with elution buffer (10 mM Tris-HCl, pH 8.0, and 150 mM imidazole). Eluted fractions were analyzed in SDS-PAGE (12%) and the fractions containing r*Lr*IL-10 were pooled together and dialyzed in 10 mM Tris-HCl, pH 7.0, using a 3.5 kDa cut-off dialysis membrane (Spectrum Laboratories, USA). The dialyzed r*Lr*IL-10 was concentrated using Amicon ultracentrifugal filter device (Millipore, USA) and stored at −20°C in small aliquots. The integrity of the purified r*Lr*IL-10 was confirmed by MALDI-TOF-MS analysis (Sandor Proteomics, Hyderabad). Pierce LAL chromogenic endotoxin quantitation kit (Thermo Scientific, USA) was used as per the manufacturer's direction to determine LPS levels in the purified r*Lr*IL-10. BCA protein estimation kit (Bio-Rad, USA) was used to determine protein concentration in different fractions as per the manufacturer's protocol. To assess the oligomeric state of the purified r*Lr*IL-10, the recombinant protein was analyzed by SDS-PAGE under both nondenaturing condition (unboiled in the absence of reducing agent) and denaturing condition (boiled in the presence of reducing agent) as detailed in the legend.

### 2.4. Generation of Polyclonal Sera against r*Lr*IL-10 in Mice

Purified r*Lr*IL-10 protein (10 *µ*g) was emulsified in Freund's complete adjuvant (1 : 1 ratio) and injected intraperitoneally (i.p.) into BALB/c mice. Preimmune sera were collected prior to immunization. The mice were bled on day 14 after immunization. First booster with the same amount of antigen emulsified in Freund's incomplete adjuvant (1 : 1 ratio) was given on day 15. Sera were prepared from the blood samples collected on days 21 and 35 after immunization. Briefly, the blood was allowed to clot for 2 h at room temperature and the serum was collected by centrifugation at 3000 ×g. The sera samples were stored at −20°C. The titer of anti-r*Lr*IL-10 was determined by ELISA.

### 2.5. Enzyme Linked Immunosorbent Assay (ELISA)

The relative titer of antibodies in the generated polyclonal antisera against r*Lr*IL-10 was determined by direct ELISA using antigen-excess assay. Purified r*Lr*IL-10 (5 *µ*g) was coated on polypropylene ELISA plate using coating buffer (0.2 M carbonate-bicarbonate buffer, pH 9.2) and incubated for 1 h at 37°C. Nonspecific sites were saturated by 5% fat-free skimmed milk in PBS-Tween 20 solution (PBST). Different dilutions of anti-r*Lr*IL-10 antisera were added with 2% BSA in PBST and incubated for 1 h at 37°C. HRP-conjugated goat antimouse IgG was then added to each well and incubated at 37°C for 1 h. Wells were thoroughly washed between successive incubations with PBST. The color was developed with orthophenylenediamine (OPD, Sigma-Aldrich Chemical Co., USA) in citrate phosphate buffer, pH 5.5 (0.5 mg/mL), containing H_2_O_2_ (1 *µ*L/mL, added just prior to use). The reaction was terminated by the addition of 2 N H_2_SO_4_ and absorbance at 490 nm was measured in a microplate reader (BioTek Power Wave XS, USA).

### 2.6. Western Blot Analysis

Western blot analysis using anti-His monoclonal antibody or anti-r*Lr*IL-10 antisera was performed essentially as described earlier [30]. Protein samples resolved on the SDS-PAGE were transferred onto the nitrocellulose membrane and the blot was then blocked with 5% nonfat milk (Difco) in phosphate buffered saline containing 0.1% Tween 20 (PBST) overnight at 4°C. This was followed by incubation with primary antibody diluted in PBST (Sigma-Aldrich Chemical Co., USA) for 1 h at room temperature, followed by three PBST washes. Secondary antibody (anti-mouse HRP, Sigma-Aldrich Chemical Co., USA) was then added (1 : 5000 dilution in PBST) to react for 1 h. After washing the blot extensively with PBST, the blot was developed by addition of 3,3′-diaminobenzidine (0.05% DAB) after addition of hydrogen peroxide in PBS (30%, 1 *µ*L H_2_O_2_/mL of DAB solution) as described earlier [30]. The reaction was terminated by washing the membrane several times with water. Alternatively, the blot was developed using Pierce ECL Western blotting substrate (Thermo Scientific, USA) and the images were acquired in a Biospectrum 500 Imaging system (UVP, Cambridge, UK) [31].

### 2.7. 2-D Gel Electrophoresis

For 2-D gel electrophoresis analysis, r*Lr*IL-10 (~250 *μ*g) was precipitated using acetone and resuspended in 0.5 mL solubilization buffer (7 M urea, 2 M thiourea, 2% w/v CHAPS, 5 mM dithiothreitol (DTT), and 1% (v/v) IPG 3–10 buffer (GE Healthcare, USA)). Samples were loaded onto 7 cm polyacrylamide IPG strips (pH 3–10 Linear, GE Healthcare, USA) by in-gel rehydration at 50 V for 11 h. Focusing was conducted at 20°C in an Ettan IPGphor (Amersham, USA) as described earlier [32]. Second dimension electrophoresis was performed by 12% SDS-PAGE analysis followed by staining with coomassie brilliant blue R-250 (Sigma-Aldrich Chemical Co., USA). The spots were excised out and analyzed by MALDI-TOF-MS for protein identification (Sandor Proteomics, Hyderabad).

### 2.8. Circular Dichroism (CD) Spectroscopy

Far-UV (200–250 nm) CD spectra of the r*Lr*IL-10 (0.2 mg/mL in 10 mM Tris-HCl, pH 7.0) at 25°C were recorded using a spectropolarimeter (JASCO J-815; path length, 0.1 cm; scan speed, 50 nm/min). Three successive spectra were accumulated and averaged followed by baseline correction. Mean residue weight ellipticities were expressed as degree × cm^2^  × dmol^−1^. Secondary structure contents of the refolded protein from the CD measurements were calculated using the K2D2 software (http://www.ogic.ca/projects/k2d2/).

### 2.9. Fluorescence Spectroscopy

A fluorescence spectrum of the r*Lr*IL-10 (0.2 mg/mL in 10 mM Tris-HCl, pH 7.0) was monitored using a spectrofluorimeter (Cary Eclipse, Varian Optical Spectroscopy Instruments, Australia). Sample was excited at a wavelength of 280 nm (slit width 10) and emission spectra were monitored at the wavelength range of 300 nm and 500 nm (slit width 10) at scan of 30 nm/min. The height at maximal emission (emission maxima) was measured. The steady state of r*Lr*IL-10 was graphically represented by plotting fluorescence intensity at 334 nm on *y*-axis and gradual increase of temperature from 20°C to 90°C at the interval of 5°C incubated for 20 min at each temperature was plotted on *x*-axis. Kinetics of r*Lr*IL-10 unfolding were represented by plotting fluorescence intensity at 334 nm versus incubation time.

### 2.10. Nitroblue Tetrazolium (NBT) Reduction Assay

To detect reactive oxygen species (superoxide anion (O^2−^), hydrogen peroxides (H_2_O_2_), and hydroxyl radicals (OH^−^) production by phagocytic cells), oxidative burst activity was measured by reduction of NBT by the method of Das et al. [33] with minor modification.

The optimum concentration of r*Lr*IL-10 required to induce NBT reduction was determined in a preliminary experiment. Heparinized blood was collected from five fish. Blood samples (100 *µ*L) from each fish (in triplicate) were treated with equal volume of PBS or r*Lr*IL-10 (1000 ng in 100 *µ*L PBS) and allowed to incubate for 1 h at 25°C. Equal volume of 0.2% NBT (Sigma-Aldrich Chemical Co., USA) was then added to the samples and incubated further for 1 h at 25°C to allow formazan granules formation. Intracellular reduction of NBT was determined by solubilizing the formazan crystals with dimethyl formamide (SRL, India), centrifugation at 2000 ×g for 5 min, and measurement of absorbance of the supernatant at 540 nm.

### 2.11. Expression Profile of Immune Related Genes in* L. rohita*

To study the immunoregulatory properties of r*Lr*IL-10, 12 rohu juveniles (weighing ~50 g) were injected with 100 ng of r*Lr*IL-10 (i.p.). Similar numbers of fishes were administered with PBS to serve as control. Spleen tissues were aseptically collected in RNAlater (Sigma-Aldrich Chemical Co., USA) from r*Lr*IL-10-administered fish at 3, 6, 12, and 24 h after injection (three fish for each time point). RNA was isolated and used for cDNA synthesis as described by Mohanty and Sahoo [34]. Semiquantitative PCR was performed for the expression of IL-1*β*, IL-6, IL-8, MHCI, MHCII, TNF*α*, and NKEF genes. Gene specific primers were obtained from Xcelris, Ahmedabad, India, based on the primer sequence information used earlier [33–35]. The constitutively expressed housekeeping gene, *β*-actin, was used both as a positive control and for normalization. To assess the level of expression of the target genes, densitometric analysis was performed using Alpha Ease FC Imaging Software (Alpha Innotech Corp., USA). The ratios of immune related genes/*β*-actin product were subsequently calculated after subtraction of the background pixel intensity for each gene of interest and used to assess the differences in expression levels between control and the various infected samples. Statistical analysis was performed applying Student's *t*-test. The* p* values below 0.05 were considered to be statistically significant.

## 3. Results 

### 3.1. Cytoplasmic Expression of* Lr*IL-10

**Figure 1:**
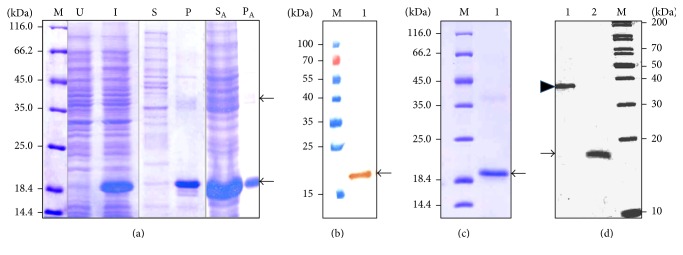
Analysis of expression of recombinant IL-10 of* L. rohita*. (a)* E. coli *BL21 (*λ*DE3) cells harboring pET*Lr*IL-10 were induced with IPTG. Lanes “U” and “I” represent total cell lysates of uninduced and induced cells, respectively. Lanes S and P represent the soluble and pellet fractions, respectively, of the induced cell lysate prepared in 10 mM Tris-HCl, pH 7.0. Lanes S_A_ and P_A_ represent the soluble and insoluble fractions, respectively, of the induced cells lysed in 10 mM Tris-HCl, pH 7.0, containing 0.5 M arginine. (b) Western blot analysis of induced cell lysate prepared in the presence of 0.5 M arginine (lane 1) using anti-His antibody. (c) SDS-PAGE (12%) analysis of purified r*Lr*IL-10 (lane 1). Arrow in Panels (a)–(c) points to r*Lr*IL-10. (d) Analysis of oligomeric state of r*Lr*IL-10. SDS-PAGE analysis of the unboiled (lane 1) and boiled r*Lr*IL-10 (lane 2), prepared in nonreducing and reducing conditions, respectively. The arrow head and arrow point to the dimeric and monomeric forms of the r*Lr*IL-10 in unboiled and boiled samples, respectively. M in all the panels indicates protein molecular weight markers.

Induction of* E. coli* BL21 (*λ*DE3) cells, harboring plasmid pET.r*Lr*IL-10, with IPTG at 37°C for 5 h resulted in high level of expression of 6x histidine-tagged r*Lr*IL-10 as a band of the expected size of ~18 kDa was seen in the induced cell lysate (Figure 1(a), lane I). Absence of this band at the same position in uninduced cell lysates indicated tight control of expression (Figure 1(a), lane U). Analysis of the soluble (S) and insoluble fractions (P) prepared by sonication of the induced cells revealed r*Lr*IL-10 to have been expressed only in the insoluble fraction. Induction with lower concentrations of IPTG (0.1 mM to 0.5 mM) or at lower temperatures (6, 15, 18, and 25°C) could not also direct the expression of r*Lr*IL-10 to the soluble fraction (data not shown). Presence of 0.5 M arginine in sonication buffer resulted in localization of the majority of the r*Lr*IL-10 in the soluble fraction (Figure 1(a), lane S_A_) with little remnants in pellet fraction (P_A_).

Detection of a single band at the expected position in Western blot analysis of the soluble fraction (obtained by sonication in lysis buffer containing 0.5 M arginine) using anti-His antibody (Figure 1(b), lane 1) confirmed the authenticity of the 6x Histidine-tagged r*Lr*IL-10.

### 3.2. Purification of r*Lr*IL-10 from Soluble Fraction

As presence of arginine in the sonication buffer hindered effective binding of the r*Lr*IL-10 with the affinity resin, arginine was removed from the soluble fraction using PD10 column (GE Healthcare, USA) prior to affinity chromatography. Using Hispur Cobalt resin-based affinity chromatography, the recombinant r*Lr*IL-10 was purified to near homogeneity (Figure 1(c), lane 1). A very faint band at ~36 kDa possibly indicated the presence of dimeric rLrIL-10.

Oligomeric state of the r*Lr*IL-10 was confirmed by SDS-PAGE analysis under nondenaturing and denaturing conditions. As evident from Figure 1(d), the unboiled r*Lr*IL-10 under nonreducing conditions showed a band of ~36 kDa corresponding to the dimeric form of the recombinant protein (lane 1). However, the r*Lr*IL-10 dimer got fully converted into ~18 kDa band corresponding to the monomeric form of the recombinant protein when the sample was analyzed under reducing conditions after boiling for 10 min (lane 2). These data suggest that the r*Lr*IL-10 exists in a dimeric state. Approximately 70 mg of the purified soluble r*Lr*IL-10 was obtained per liter of* E. coli* culture at shake flask level. LPS concentration in the purified r*Lr*IL-10 was determined to be 0.015 pg/*µ*g of purified protein, which is within the permissible limit of the LPS for immunization studies.

**Figure 2:**
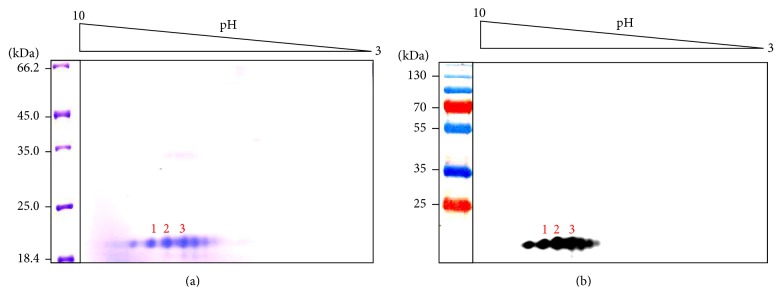
Two-dimensional gel electrophoresis of the purified r*Lr*IL-10. The purified r*Lr*IL-10 was resolved by isoelectric focusing using carrier ampholytes (pH 3–10, shown on top) followed by SDS-PAGE (12%). Panel (a) shows coomassie brilliant blue stained gel. The spots 1–3 were subjected to MALDI-TOF-MS (shown in Figures  1–3 in Supplementary Material). (b) Western blot analysis of identical gel shown in Panel (a) using anti-r*Lr*IL-10 antibodies. kDa indicates migration of the protein markers.

Two-dimensional gel electrophoresis of the r*Lr*IL-10 revealed the presence of 6 spots (Figure 2). The 6 spots were distributed like a string forming the so-called “train” between pI values of 7 and 9.5, indicating the presence of several isoforms of r*Lr*IL-10 differing in their pIs. Western blot analysis of the r*Lr*IL-10, resolved on 2-D gel, with anti-r*Lr*IL-10 antibody confirmed the spots to be isoforms of r*Lr*IL-10 (Figure 2(b)). MALDI-TOF-MS of three of the spots (Figure 2(a), numbered 1–3) resulted in generation of the individual peptide with the monoisotopic mass in the range of 567.244–4973.573 (spot 1), 524.007–4973.538 (spot 2), and 534.175–4098.731 (spot 3) for three spots, respectively (supplementary data, Panel A in Figures S1–S3 in Supplementary Material available online at http://dx.doi.org/10.1155/2016/3962596). Search of the sequences obtained from the peptide mass fingerprinting (PMF) of the digested protein using Mascot search engine identified and confirmed these spots as IL-10 protein from* L. rohita* (Panel B in supplementary data, Figures S2–S4), thus confirming these spots to be isoforms of r*Lr*IL-10.

### 3.3. Biophysical Characterization of r*Lr*IL-10

**Figure 3:**
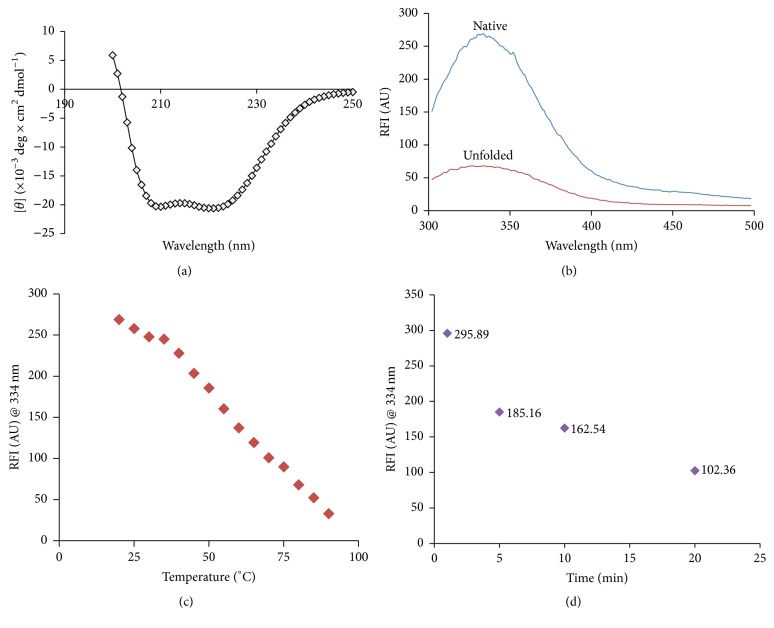
(a) Circular dichroism (CD) spectra of the soluble r*Lr*IL-10 in the far-UV regions (200–250 nm). CD values are expressed as [*θ*], mean residue mass ellipticity in units of degree × cm^2^ dmol^−1^. (b) Intrinsic fluorescence spectra of the purified r*Lr*IL-10 under native (room temperature) and unfolded (at 80°C) states. The samples were excited at 280 nm and emission spectra were recorded from 300 nm to 500 nm. RFI (AU) denotes relative fluorescence intensity in arbitrary units. (c) Temperature kinetics of unfolding of r*Lr*IL-10. Emission spectra of the r*Lr*IL-10 incubated for 20 min at different temperatures were recorded. Relative fluorescence intensity at 334 nm is plotted as a function of temperature. (d) Time kinetics of unfolding of purified r*Lr*IL-10 at 60°C. The purified r*Lr*IL-10 was incubated at 60°C for different time points and fluorescence spectra were recorded. The figure shows fluorescence intensity in arbitrary units (AU) at 334 nm at different time points.

The secondary structure of r*Lr*IL-10 was analyzed by far-UV circular dichroism spectroscopy. As evident from Figure 3(a), the r*Lr*IL-10 exhibited predominantly *α*-helical structure with characteristic double minima in ellipticity at 208 nm and 222 nm. The spectra were analyzed for secondary structure prediction using K2D2 software. The *α*-helical content of the r*Lr*IL-10 was determined to be 91.67% whereas *β* strand was found to be only 0.3%.

Fluorescence spectroscopy of the r*Lr*IL-10 was performed to analyze tertiary structure of the protein. An excitation wavelength of 280 nm was used to excite all 8 Tyr residues. The r*Lr*IL-10 gave emission maxima at 334 nm indicating intact tertiary structure (Figure 3(b)). A significant decrease in the emission maxima was observed when the protein was heated at 80°C indicating loss of tertiary structure. In order to study the steady state of unfolding of the r*Lr*IL-10, r*Lr*IL-10 was incubated for 20 min at each temperature followed by fluorescence spectroscopy. This analysis showed consequent decrease in fluorescence intensity at 334 nm with gradual increase of temperature (Figure 3(c)). When the protein was heated at a fixed temperature (60°C) for different time periods, kinetics of r*Lr*IL-10 unfolding showed gradual decrease in fluorescence intensity at 334 nm with increase in incubation time (Figure 3(d)).

### 3.4. Functional Characterization of r*Lr*IL-10

#### 3.4.1. Effect of r*Lr*IL-10 on Superoxide Production

**Figure 4:**
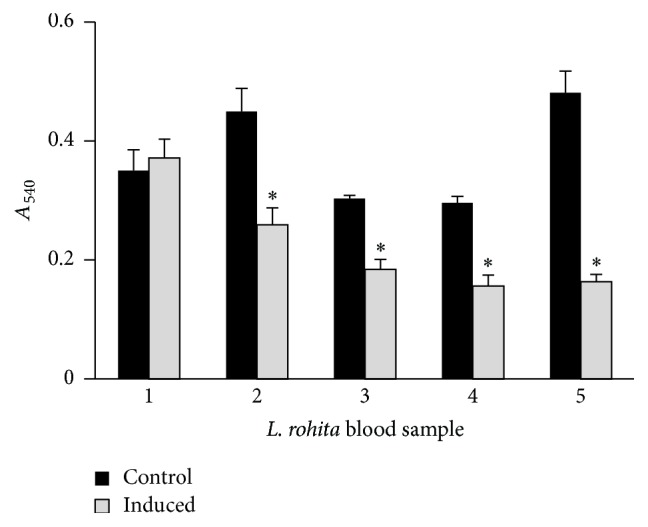
Effect of r*Lr*IL-10 on phagocytic activity of peripheral blood cell. Peripheral blood cells (in triplicate) from 5 different fishes were treated with PBS (control) or r*Lr*IL-10 (10 *μ*g/mL). Phagocytic activity was measured by NBT uptake and formazan crystals formation. Data represent mean ± SD. “*∗*” denotes statistically significant difference (*p* ≤ 0.05) in phagocytic activity of blood cells from the individual fish treated with r*Lr*IL-10 in comparison to control.

In order to establish functional activity of the r*Lr*IL-10 in modulating superoxide production, peripheral blood cells of* L. rohita* were treated with different concentrations of the recombinant protein and the optimum concentration of r*Lr*IL-10 to induce NBT reduction was found to be 10 *µ*g/mL (supplementary data, Figure S4). Addition of r*Lr*IL-10 in NBT assay clearly showed a significant decrease in the phagocytic activity in all five samples as compared to control ones (Figure 4).

#### 3.4.2. Effect of r*Lr*IL-10 Administration on Expression Profile of Immune Related Genes in* L. rohita*

**Figure 5:**
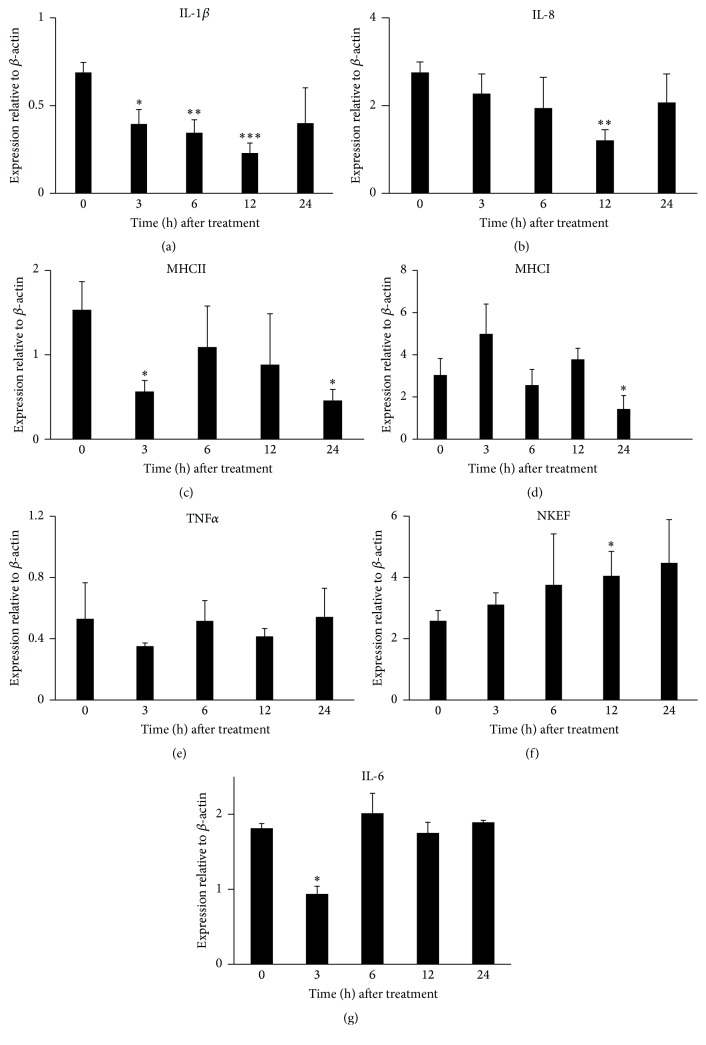
Analysis of expression of different immune genes in spleen of* L. rohita* administered with r*Lr*IL-10 at different time points after treatment. The bars represent expression of the respective gene relative to *β*-actin gene at respective time point. Bars represent mean values (±SD) of three samples. Statistically significant expression of gene relative to control is denoted by “*∗*” at *p* ≤ 0.05; “*∗∗*” at *p* ≤ 0.005; “*∗∗∗*” at *p* ≤ 0.001.

The r*Lr*IL-10 was found to effectively downregulate the expression of the proinflammatory cytokines (IL-1*β*, IL-8, MHCII, and MHCI) in* L. rohita, *whereas an increase in the expression of NKEF level was noticed (Figure 5). IL-1*β* expression started to decline from three hours onwards and remained significantly lower till 12 h (*p* ≤ 0.005) when compared to 0 h. Although there was a slight rise in IL-1*β* expression at 24 h, when compared to its levels at 12 h, it still remained lower than that at 0 h (Figure 5(a)). Similarly, a decline in IL-8 levels was observed from 3 h onwards which became significantly lower at 12 h (*p* ≤ 0.005), followed by an increase at 24 h (Figure 5(b)). The r*Lr*IL-10 resulted in a significant decrease in MHCII expression at 3 h (*p* ≤ 0.05) and 24 h (*p* ≤ 0.005) (Figure 5(c)), whereas MHCI expression levels (Figure 5(d)) were significantly lower at 24 h (*p* ≤ 0.05) after an initial rise in its level at 3 h. No statistically significant change in TNF*α* expression was noted at any of the time points (Figure 5(e)). Unlike these molecules, a gradual increase in the expression of natural killer enhancing factor (NKEF) was observed from 3 h onwards (Figure 5(f)). The NKEF levels were significantly higher at 12 h (*p* ≤ 0.05) when compared to its expression levels at 0 h. A significant decline (*p* ≤ 0.05) in the IL-6 levels was observed at 3 h after rLrIL-10 treatment in comparison to the control. At subsequent intervals, no significant change in its levels was observed (Figure 5(g)).

## 4. Discussion

IL-10 is a main anti-inflammatory cytokine that is produced by a variety of immune cells. Cells of monocytic/macrophage lineage are primary targets of IL-10 and its pleiotropic role is well documented [36]. Cloning and characterization of IL-10 gene from different species revealed the existence of interspecies variation and heterogeneity at structural and functional levels. Also, expression patterns of IL-10 showed great variation between/within fish and other vertebrates. While IL-10 has been characterized in number of bony fishes, structural and functional characterization of IL-10 from* L. rohita*, an economically important freshwater carp, has not been investigated.

The present study describes the expression, purification, and biophysical and functional characterization of recombinant IL-10 of* L. rohita.* Overexpression of r*Lr*IL-10 in* E. coli *BL21 (*λ*DE3) resulted in inclusion bodies of mis-folded and aggregated protein. As refolding and subsequent purification from inclusion bodies are difficult and produce low yields of bioactive protein, an effective strategy to obtain soluble r*Lr*IL-10 was used by including L-arginine (0.5 M) in the sonication buffer. Inclusion of arginine in the culture medium has been reported to result in expression of proteins in their active form [37]. While lower concentrations of arginine in solvents are used for refolding of the proteins expressed as inclusion bodies, extraction of active soluble folded proteins from insoluble pellets obtained by lysis of* E. coli* cells has been achieved by using higher concentrations of arginine [38]. Addition of varying concentrations of arginine to the culture medium as reported by Schäffner et al. [37] resulted in low cell density and thus affected the yield of soluble protein. In the present study, inclusion of arginine during sonication resulted in directing the protein in soluble fraction, thus significantly reducing the amount of arginine that would otherwise be required if added in culture medium. High yield of soluble r*Lr*IL-10 was thus recovered using single step purification from heterologous expression system. Authenticity of the r*Lr*IL-10 was confirmed by MALDI-TOF-MS, where mass of the peptide generated by tryptic digestion matched with those of IL-10 protein sequence in the database.

CD spectroscopy of the r*Lr*IL-10 showed predominantly alpha helical secondary structure of the r*Lr*IL-10, which is in conjunction with crystal structure of both the dimeric forms and engineered monomeric form of human IL-10 [39, 40]. Changes in the conformation of the r*Lr*IL-10 at neutral pH as a function of temperature indicated gradual loss in the helical content with an increase in temperature thus indicating unstable structure of IL-10.

Tertiary structure of the r*Lr*IL-10 was assessed by fluorescence spectroscopy. Protein sequence of mature IL-10 contains six phenylalanine residues and eight tyrosine residues with intrinsic fluorescence properties. However, for tertiary structure characterization, we only measured Tyr fluorescence because its quantum yield was high enough to give a good fluorescence signal. The native folded state of r*Lr*IL-10 contained tyrosine residues within the core of the protein, whereas in a partially folded or unfolded state they become exposed to solvent. In a hydrophobic environment tyrosine yielded a high quantum and hence high fluorescence intensity. Thermal denaturation resulted in unfolding of the protein thus exposing the Tyr residues to a hydrophilic environment reflected by a decreased fluorescence intensity. Increase in temperature resulted in loss of tertiary structure as well.

Dimeric nature of human IL-10, demonstrated by its crystal structure, has been shown to be essential for its functional activity [39]. Human IL-10 consists of two 160 amino acid residue-long polypeptide chains [41]. In humans, IL-10 dimer is thought to be the result of an evolutionary mechanism of protein oligomerization often referred to as 3D domain swapping [42]. This suggests that IL-10 evolved from a monomeric protein by exchanging structural domains (*α*-helices E and F for IL-10) with another monomer to create the dimer [40]. Similar to humans, biologically active recombinant IL-10 of viral and murine organisms has also been shown to exist as dimer [43]. Oligomeric state of the r*Lr*IL-10 was confirmed by Western blot analysis of r*Lr*IL-10 using unboiled samples under nonreducing conditions together with denatured samples under reducing conditions. Our results show that r*Lr*IL-10 exists as a dimer as reported for its homologues from other species. The dimeric state of IL-10 has been reported to be concentration dependent and an increase in dimeric forms with an increase in concentration of recombinant viral and murine IL-10 has been reported [43].

Two-dimensional gel electrophoresis showed presence of several isoforms of r*Lr*IL-10 which differed in their pI (pI range 7.5 to 9.5). Immunoblotting and MALDI-TOF-MS confirmed these to be r*Lr*IL-10. Theoretical pI of the histidine-tagged r*Lr*IL-10 was determined to be 7.96. The slight charge microheterogeneity in the r*Lr*IL-10 could arise due to posttranslational modification, deamidation of the protein during purification or storage, and differential oxidation-reduction state [44–46].

IL-10 plays a major role in withstanding inflammation through regulating proliferation, differentiation, and activation of immune cells [10]. The biologically active r*Lr*IL-10 was shown by inhibition of superoxide production as a measure of phagocytosis and its pleiotropic immunoregulatory properties. Phagocytosis is accompanied by an oxidative burst with an increase in the production of reactive oxygen species [47]. Our results are in agreement with earlier reports put forth by Bogdan et al. [16], who reported recombinant mouse IL-10 to inhibit primed peritoneal macrophages function by markedly decreasing the release of reactive oxygen species. A marked reduction in production of reactive oxygen species by recombinant goldfish IL-10 has also been reported in monocytes primed with* Aeromonas salmonicida* and recombinant goldfish IFN-*γ* [13]. In the present study, a significant decrease in superoxide production activity of leucocytes of* L. rohita *possibly resulting from inhibitory effect of IL-10 on H_2_O_2_ production was observed even without priming. Decreased phagocytic activity was indeed r*Lr*IL-10 induced as no effect was observed when the treatment was given with heat-denatured r*Lr*IL-10 (data not shown). However, Piazzon et al. [26] did not observe any effect of recombinant carp IL-10 on free radicals production in unstimulated carp neutrophils and macrophages. It is to be noted that the r*Lr*IL-10 concentration used in the present study was much higher (corresponding to 10 *µ*g/mL) than that reported used by Piazzon et al. [26] for carp IL-10 (0.005–0.5 U/mL) or by Grayfer et al. [13] for recombinant goldfish IL-10 (up to 1 *µ*g/mL) using primed cells. Thus, with higher concentrations the effect could be seen even in unprimed cells.

Functional analysis of the r*Lr*IL-10* in vivo* (administration in* L. rohita*) showed that it downregulated most of the proinflammatory cytokines and upregulated natural killer enhancing factor in spleen. These results are consistent with anti-inflammatory function reported for IL-10 homologues from other species [6, 7, 13, 20]. This negative regulation is mediated by the JAK1/STAT3 pathway, and STAT3 is the primary mediator of IL-10 effects [48]. Downregulation of IL-1*β* expression as early as 3 h after r*Lr*IL-10 treatment is expected as IL-1*β* is one of the early response proinflammatory cytokines that enables organisms to respond to infections, inducing an inflammatory cascade, along with other defensive responses. In goldfish also recombinant IL-10 administration resulted in decreased levels of IL-1*β* [13]. Seppola et al. [23] reported opposite patterns of expression of IL-10 and IL-1*β* during different stimulations in spleen tissue of Atlantic cod. In this report, a decrease in IL-1*β* with an increase in IL-10 expression and vice versa confirms the anti-inflammatory effect of IL-10. IL-6 is a pleiotropic cytokine with a central role in the host defense. Although it had been described as a proinflammatory cytokine, its anti-inflammatory and immunosuppressor properties have also been reported [49]. IL-10 has generally been reported to downregulate IL-6 expression together with other proinflammatory cytokines such as IL-1 and TNF*α* [15]. LPS-mediated production of IL-6 has also been reported to be inhibited by IL-10 and this effect of the IL-10 is blocked by STAT3 [48]. Thus, a significant decline in IL-6 transcript levels 3 h after rLrIL-10 exposure in the present study is in agreement with the earlier reports [15, 48, 50] and possibly points towards its pronounced anti-inflammatory role rather than proinflammatory one.

IL-10 has been known to inhibit antigen specific T cell responses by regulating expression of MHCII on the surface of monocytes [51]. Downregulation of MHCII by r*Lr*IL-10 observed in the present study is in line with the reports put forth by Lin et al. [52], who showed that the rIL-10b isoform produced by human cytomegalovirus inhibited MHCII expression on granulocyte macrophage progenitors. Earlier studies have also shown that mouse cytomegalovirus infection resulted in premature and transient activation of host IL-10 very early during infection resulting in a significant and selective reduction of MHCII expression on cell surface [53]. Downregulation of MHCII expression by r*Lr*IL-10 in* L. rohita *showed that the function of IL-10 on regulation of antigen presentation has remained conserved across species. Although a generalized downregulation of MHCII was noticed in the present study after r*Lr*IL-10 exposure in* L. rohita*, a significant decline at 3 and 24 h might be indicative of differential transient expression at individual level. Earlier studies have established that the inhibition of production of reactive oxygen species is directly linked to inhibition of TNF*α* synthesis [18]. However, in our studies, though we observed inhibition of phagocytic activity of peripheral blood cells of* L. rohita in vitro*, TNF*α* expression in spleen of treated fishes was not significantly altered at all the study intervals when compared to 0 h. However, a decrease of ~40% was noted at 3 h. Downregulation of proinflammatory cytokines by IL-10 indicates its central role in protection of cells against excessive immune responses and excessive tissue damage. The activation of natural killer cells might contribute to the clearance of the pathogen and facilitate antigen acquisition from dead cells for cross-priming by activated APCs, providing a link between the innate and the adaptive immune responses [54, 55].

## 5. Conclusion

Thus, the present study provides comprehensive biophysical and functional characterization of IL-10 from* L. rohita, *a freshwater Indian major carp. Soluble r*Lr*IL-10 thus produced in large amounts using heterologous expression system exhibited structural (helical and oligomeric state) and biological properties (anti-inflammatory) reported for IL-10 from other species and have remained conserved. The present study will help in better understanding of the IL-10 modulated immune responses in* L. rohita*. As IL-10 of* L. rohita *is established to generate an anti-inflammatory response, application of the r*Lr*IL-10 to control excessive immune response in fish can be explored.

## Supplementary Material

Recombinant IL-10 of *Labeo rohita* was resolved by 2-dimensional gel electrophoresis and the spots numbers 1-3 shown in Fig. 2A were subjected to MALDI-TOF-MS analysis for protein identification. For this, the spots were carefully excised out from the polyacrylamide gel using a sterile salpel and subjected to trypsin digestion. The tryptic digests were then by MALDI-TOF-MS system from Bruker Daltonik GmbH, Bremen, Germany at Sandor Proteomics, Hyderabad. MASCOT search engine (() was used to compare the masses of tryptic fragments thus obtained with the masses of identified peptides available in the NCBI protein database for protein identification.
