# Supplementary material for: Structural and Functional Characterization of Recombinant Interleukin-10 from Indian Major Carp Labeo rohita
Source: J Immunol Res. 2016 Sep 1;2016:3962596. doi: 10.1155/2016/3962596 (PMC5023823; doi:10.1155/2016/3962596)
Supplement: Supplementary file 1 — Recombinant IL-10 of Labeo rohita was resolved by 2-dimensional gel electrophoresis and the spots numbers 1-3 shown in Fig. 2A were subjected to MALDI-TOF-MS analysis for protein identification. For this, the spots were carefully excised out from the polyacrylamide gel using a sterile salpel and subjected to trypsin digestion. The tryptic digests were then by MALDI-TOF-MS system from Bruker Daltonik GmbH, Bremen, Germany at Sandor Proteomics, Hyderabad. MASCOT search engine (() was used to compare the masses of tryptic fragments thus obtained with the masses of identified peptides available in the NCBI protein database for protein identification. [file 3962596.f1.pdf]

A

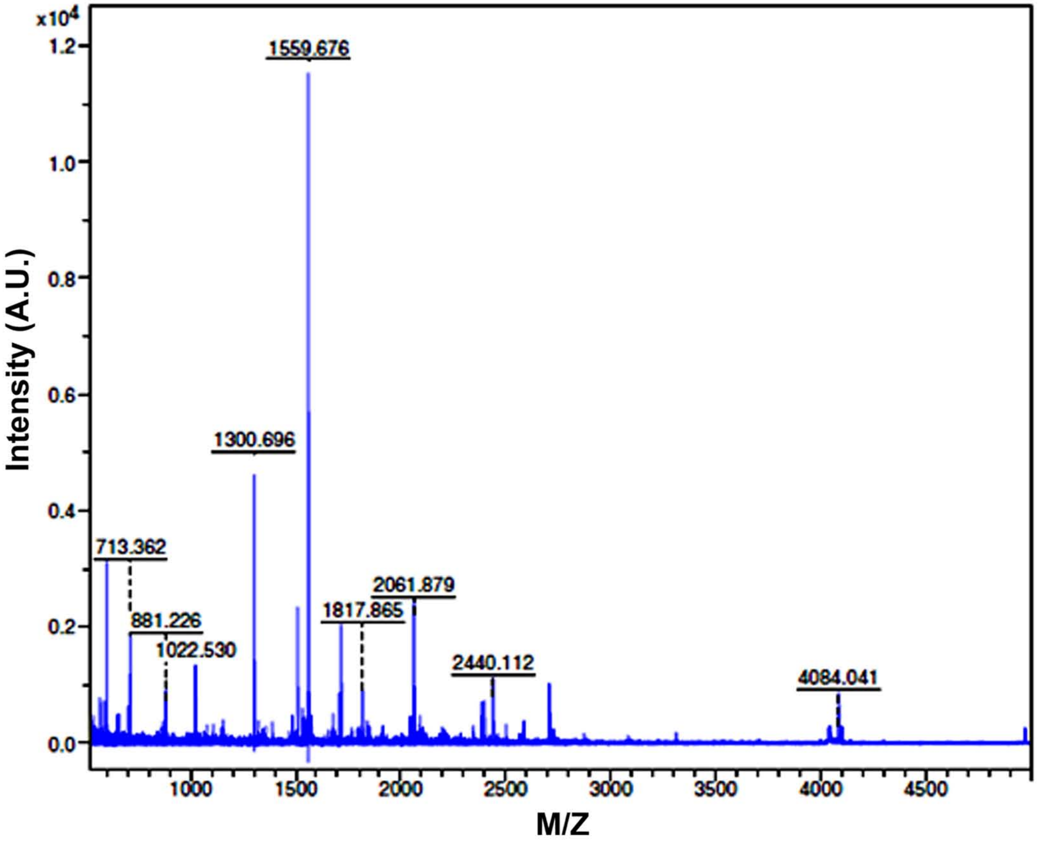

B

Sequence data:		
Interleukin - 10 *Labeo rohita*	Sequence Coverage MS:	48 %
Score: 96	pI (isoelectric point): 8.11	Mass: 21.314 kDa

10	20	30	40	50
MIFTGVILSS	LVMLLLSDSA	QCRRVDCKSD	CCSFVEGFPV	RLKELRSAYR
60	70	80	90	100
EIQRFYESND	DMEPLLNENV	QQNINSPYGC	HVMNEILRFY	LDTILPTAVQ
110	120	130	140	150
KSHLHSKTPI	DSIGNIFQDL	KRDMLKCKNY	FSCQNPFELA	SIKNSYEKMK
160	170	180		
EKGVSKAMGE	LDMLFKYIEQ	YLTSKRVKHL		

Fig. S1(A) Mass spectrum analysis report of the tryptic digest of the spot 1 of the r*Lr*IL-10 separated by 2D (shown in Fig. 2A). (B) The identified protein, score, amino acid sequence coverage and the number of identified peptides are shown. The matched peptide ions in the IL-10 sequence (shown in red) are shown in bold and underlined.

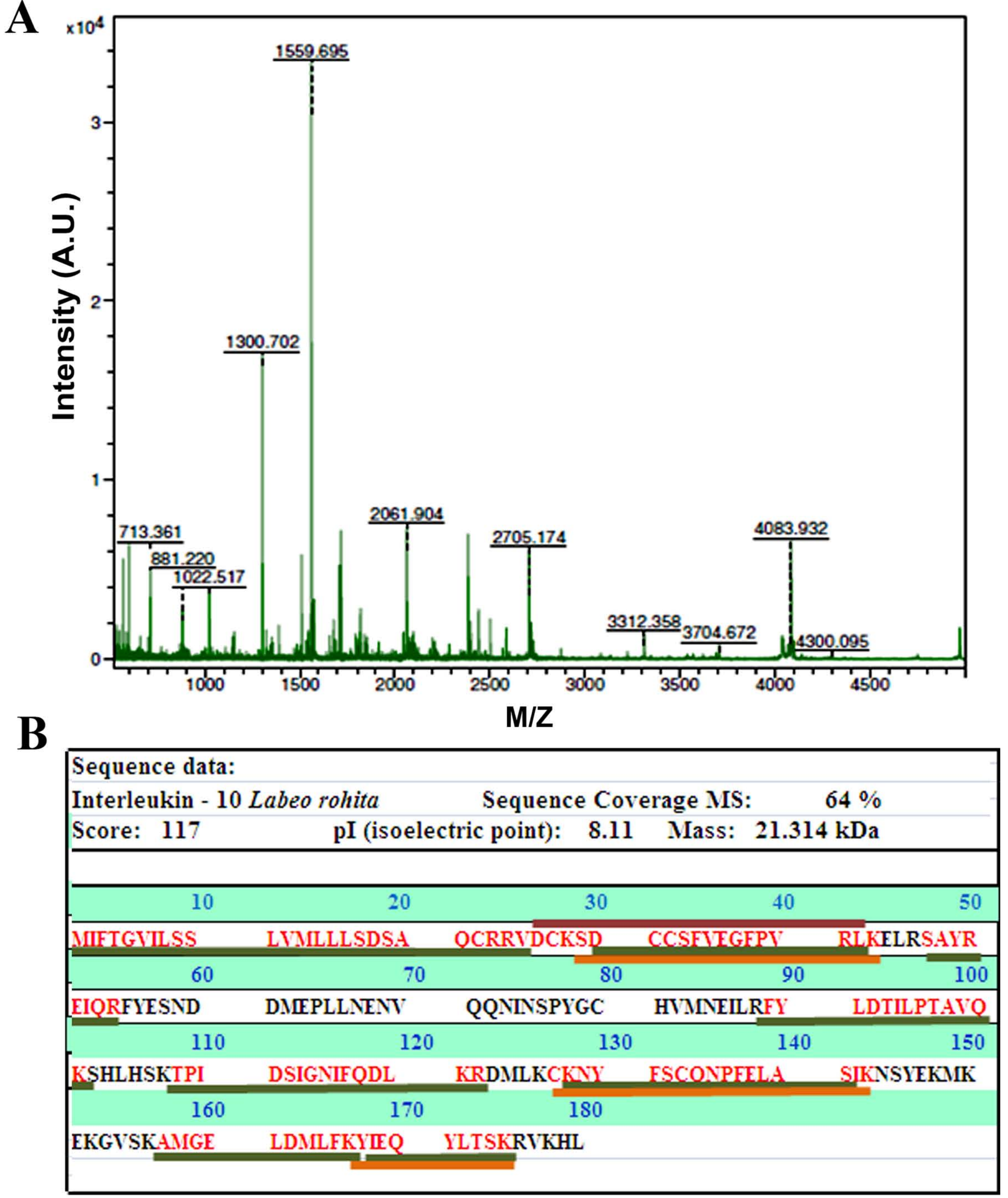

Fig. S2(A) Mass spectrum analysis report of the tryptic digest of the spot 2 of the r*Lr*IL-10 separated by 2D (shown in Fig. 2A). (B) The identified protein, score, amino acid sequence coverage and the number of identified peptides are shown. The matched peptide ions in the IL-10 sequence (shown in red) are shown in bold and underlined.

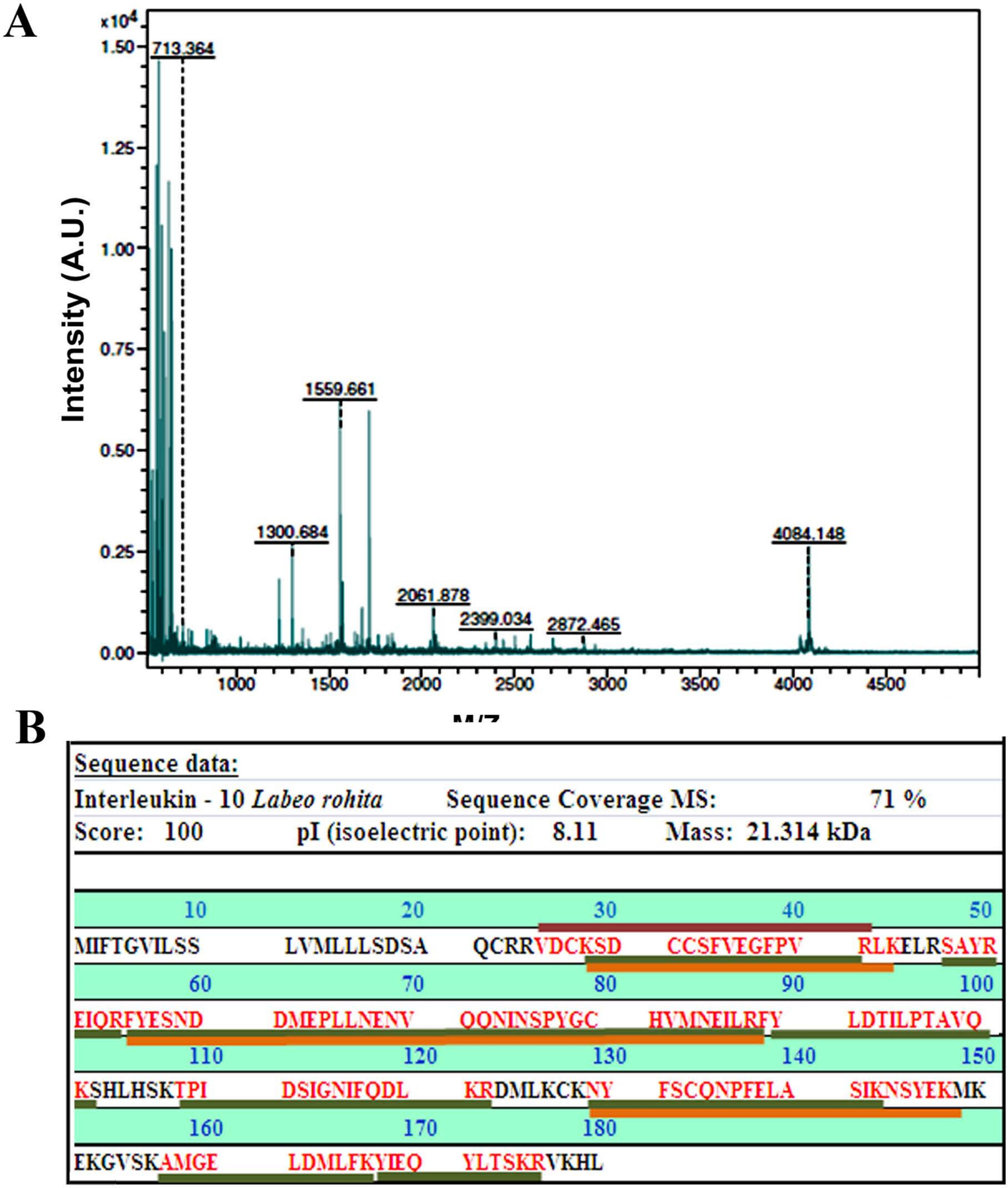

Fig. S3 (A) Mass spectrum analysis report of the tryptic digest of the spot 3 of the r*Lr*IL-10 separated by 2D (shown in Fig. 2A). (B) The identified protein, score, amino acid sequence coverage and the number of identified peptides are shown. The matched peptide ions in the IL-10 sequence (shown in red) are shown in bold and underlined.

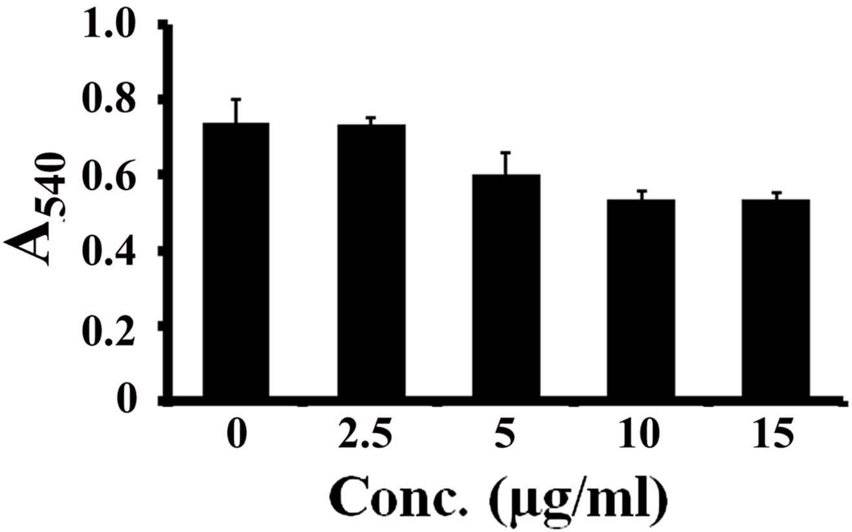

Fig. S4. To determine optimum concentration of rlrIL-10 required to induce NBT reduction, heparinized blood collected from five fish was pooled. It was then distributed into fifteen tubes containing 100 µl of blood in each tube. Three tubes were considered as negative control and were treated with corresponding volume of 1x PBS. To the remainder 12 tubes, four different concentrations of rLrIL-10 was added (three tubes each). The tubes were further incubated for 1 h at 25 °c. To each tube, NBT (0.2%, Sigma-Aldrich Chemicals Co., USA) was added in 1:1 ratio, mixed well and allowed to incubate further for 1 h at 25 °c. Further, 50 µl of this reaction mixture was transferred to glass centrifuge tube and 1 ml of dimethyl formamide (SRL, India) was added to it to solubilize the reduced formazan granule. The product was centrifuged at 2000 × g for 5 minutes and NBT reduction was measured in the supernatant of all samples at 540 nm. The data represent mean + S.D. for analysis conducted in triplicate.
